# The complete nucleotide sequence of the mitochondrial genome of *Calliphora chinghaiensis* (Diptera: Calliphoridae)

**DOI:** 10.1080/23802359.2016.1174088

**Published:** 2016-06-20

**Authors:** Yiling Chen, Xian Shi, Diyan Li, Binlong Chen, Pu Zhang, Nan Wu, Zhongxian Xu

**Affiliations:** Farm Animal Genetic Resources Exploration and Innovation Key Laboratory of Sichuan Province, Sichuan Agricultural University, Ya’an, PR China

**Keywords:** *Calliphora chinghaiensis*, mitochondrial DNA, start codon, termination codon

## Abstract

The complete mitogenome genome of *Calliphora chinghaiensis* (Diptera: Calliphoridae) is determined in this study. Mitochondrion of *C. chinghaiensis* is 15,269 bp long. It forms a circular DNA molecule that includes 37 typical animal mitochondrial genes and an A + T-rich region. All protein-coding genes are initiated by ATN start codon, except for *cox1,* which uses TCG as its start codon. 11 protein-coding genes stop with the termination codon TAN, while other protein-coding genes use CTT and AGT (*cytb*) as termination codon, respectively. Furthermore, the largest non-coding A + T-rich region with a length of 442 bp is at the end of *rrns*. The mitochondrial genome of *C. chinghaiensis* has been completely sequenced for the first time in this study.

Mitochondrial DNA (mtDNA) has been widely used in phylogenetic research studies, phylogeography, population structure and dynamics, and molecular evolution (Boore 1999; Shi et al. 2015). There is no complete mitochondrial genome of *Calliphora chinghaiensis* (Diptera: Calliphoridae) has been reported by far. In 1926, the mitochondrial genome of *C. chinghaiensis* was primitively named. Usually, *C. chinghaiensis* inhabited in mountains over 3000 m and distributed at Qinghai, Sichuan, Yunnan and Tibet in China (Hu et al. 2007). Specimens of *C. chinghaiensis* (specimen voucher SIE32066838) were collected form Shangri-la, Yunnan Province, China (N: 26°–34°, E: 94°–102°). They were identified as *C. chinghaiensis* through external morphology. Our DNA extraction method followed direction of DNA extraction kit by DNeasy Blood & Tissue Kit (Sangon Biotech, China). The complete mitochondrial genome of *C. chinghaiensis* (Diptera: Calliphoridae) has been submitted to GenBank under accession no. KT936147.

The length of complete mitochondrial genome of *C. chinghaiensis* is 15,269 base pairs (bp), with the overall A + T content of 76.73% (A = 39.25%, G = 9.95%, T = 37.48% and C = 13.28%). The genome encodes the same set of 37 genes including 13 protein-coding genes (PCGs), 22 transfer RNAs (tRNAs) and two ribosomal RNAs (rRNAs) (Clary & Wolstenholme 1987). In addition, the complete mitochondrial genome contains a non-coding A + T-region (D-loop) related to the regulation of transcription and the control of replication (Chang & Clayton, 1984; Shadel & Clayton, 1993). All the PCGs initiate with an ATN start codon (*nd2, nd3, nd5 nd6* and *cox3* start with ATT; *nd4, nd4l, cox2, cytb* and *atp6* start with ATG; *atp8* starts with ATC and *nd1* starts with ATA) except for *cox1*, which initiates with TCG start codon. The TCG as a serine is assigned as the *cox1* start codon, since the hexanucleotide ATTTAA participating initiation signalling is found next to a TCG codon (Junqueira et al. 2004). Therefore, the TCG for a serine has been assigned as the cox1 start codon. The hexanucleotide signals were detected in mosquitoes (Beard et al. 1993; Mitchell et al. 1993). Eight PCGs (*nd1, nd2, nd3, nd4l, nd6, cox1, cox3* and *atp8*) contain the typical termination codon TAA, one gene (*cytb*) stops with AGT, one gene (*cox2*) terminates with CTT. While *nd4, nd5* and *atp6* have a stop codon of TAT.

Like other insect tRNAs, the mitochondrial genome of *C. chinghaiensis* contains 22 transfer RNA genes, ranging from 64 to 72 bp, has a typical cloverleaf structure. The large mitochondrial rRNA subunit (rrnL) is 1328 bp long with an A + T content of 81.93%, while the small subunit (rrnS) has a total length of 785 bp, with an A + T content of 78.34%. The largest non-coding A + T-rich region is 442 bp long, which is shorter than the rrnS (785 bp). The A + T content is up to 84.16%, the highest of any region in the whole genome was believed to participated in the regulation of transcription and control of DNA replication (Zhang & Hewitt 1997). A neighbour-joining (NJ) tree of 16 Calliphoridae and six outgroups (R. *pernix*, *D. platura*, *H. irritans irritans*, *S. portschinskyi*, *S. crassipalpisa*, and *E. sorbillans*) based on 13 protein-coding genes is constructed by MEGA6 (Tamura et al. 2013) with the Poisson model (Figure 1). The NJ bootstrap for 1000 replicates was indicated in each node. The evolutionary position of *C. chinghaiensis* was showed in the NJ tree. Except *C. chinghaiensis*, all other mitochondrial genomes were obtained from NCBI.

**Figure 1.**
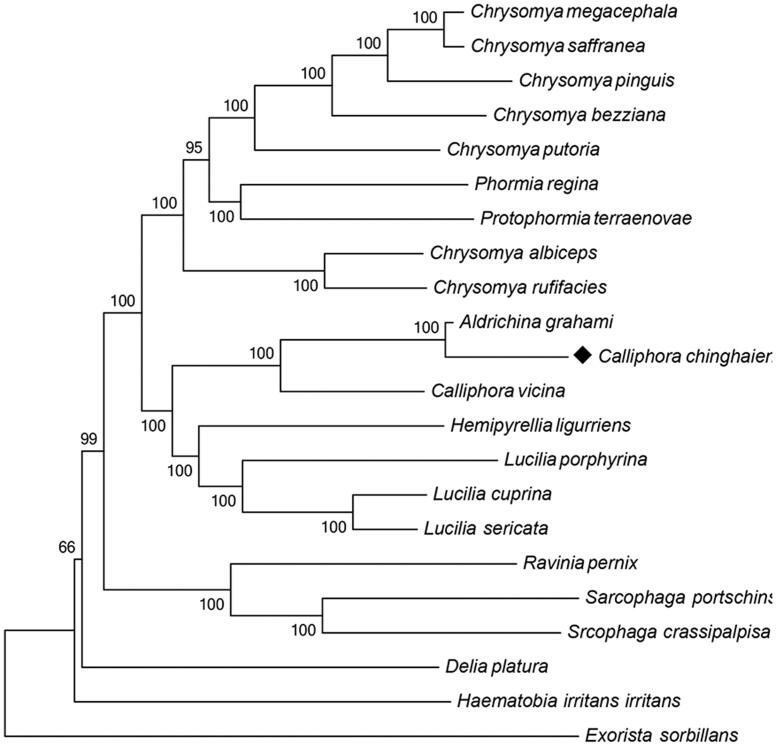
Evolutionary relationships of 16 Calliphoridae and 6 outgroup species. The mitochondrial genome of this study has been marked as black. GenBank IDs: *A. gra*hami, KP872701.1; *C. albiceps*, JX913736.1; *C. bezziana*, JX913737.1; *C. chinghaiensis*, KT936147; *C. putoria*, AF352790.1; *C. vicina*, JX913760.1; *C. megacephala*, JX913739.1; *C. saffranea*, JX913742.1; *C. pinguis*, KM244730.1; *C. rufifacies*, JX913740.1; *H. ligurriens*, JX913759.1; *P. regina*, KC005712.1; *P. terraenovae*, JX913743.1; *L. sericata*, JX913756.1; *L. cuprina*, JX913749.1; *L. porphyrina*, JX913758.1; *R. pernix*, KM676414.1; *D. platura*, KP901268.1; *H. irritans irritans*, DQ029097.1; *S. portschinskyi*, KM287570.1; *S. crassipalpisa*, KP861920.1; *E. sorbillans*, HQ322500.1.
